# Understanding agricultural land leasing in Ireland: a transaction cost approach

**DOI:** 10.1186/s40100-023-00254-x

**Published:** 2023-06-08

**Authors:** Laura Onofri, Samuele Trestini, Fateh Mamine, Jason Loughrey

**Affiliations:** 1 Department of Land, Environment, Agriculture and Forests, University of Padova, Padua, Italy; 2 French National Institute for Agriculture, Food, and Environment (INRAE), Villeneuve-d’Ascq, France; 3 Ireland Agricultural and Food Development Authority, Waterford, Ireland

**Keywords:** Land lease contract, Transaction cost economics, Two-stage least squares, Contract duration

## Abstract

Formal written land leasing contracts offer an alternative to land purchase for those farmers wishing to expand their land area and provide greater security relative to informal short-term rental agreements and are particularly important for beginning farmers with resources insufficient to purchase land. Formal land leasing contracts vary in terms of their duration, but there is limited understanding about the determinants of contract duration in developed countries. In this research, we use econometric techniques and transaction level data to explore the determinants of duration for agricultural land lease contracts for two regions in Ireland. Under the transaction cost economics approach, the research explores the role of legal status, price and non-price conditions in influencing the contract duration. Results indicate that the legal status of the tenant is a significant factor in influencing the duration. Provisions such as break clauses appear positively related to duration and confirm the theoretical expectation that long-term contracts create a demand for processes that enable adaptation over the course of long-term exchange.

## Introduction

Patterns of land tenure can change due to various reforms or events. Large-scale changes in the pattern of land tenure have attracted more attention in the econometric literature relative to moderate changes (Vranken and Swinnen 2006; Deininger et al 2012; Van Landeghem et al 2013). A recent moderate but significantly enduring increase in agricultural land leasing activity is evident in Ireland (Geoghegan et al 2021) and is largely attributed to the expansion of the dairy sector in the aftermath of milk quota abolition in 2015 (Bradfield et al 2020). The rise in rental activity appears dominated by the growth of medium- and long-term land lease contracts, but this increase is occurring from a relatively low base. Ireland has the lowest rental share in European agriculture (Swinnen et al 2016). Agricultural land rental agreements in Ireland have traditionally been informal and short term in design with few non-price provisions considered (O’Neill and Hanrahan 2012; Geoghegan et al. 2018).

In Ireland, a variety of activities contribute to the demand for agricultural land including livestock production, cereal production, forestry and other rural land use activities. The land sales market in Ireland is relatively thin (Loughrey et al 2020), and land rental transactions therefore provide an alternative for dairy farmers and other tenants wishing to expand their land area. Formal land leasing contracts are more prevalent in other Western European countries including France and Belgium where long-term land leases are the dominant form of land tenure (Adenuga et al 2021). The land rental share has remained close to 70 per cent in Belgium for over 150 years. In France, the dominance of land rental is more recent with the rental share rising from 44 per cent in 1950 to 75 per cent in 2010 (Swinnen et al 2016).

Land rental activity varies across Europe with relatively high rental shares (over 50 per cent) in Germany, the Czech Republic and Slovakia and relatively low rental shares (less than 35 per cent) in Poland, Portugal and Greece (Loughrey et al. 2019). As in the case of Ireland, a recent increase in land rental market activity is observed in Denmark and Italy (Eurostat 2020). In Norway, the land rental activity has expanded over a longer period of time (Forbord 2014). Sckokai (2021) attributes the rise in rental activity in Italy to landowner decision-making in the presence of stable land values following the financial crisis. Sckokai (2021) concludes that the deregulation of the land rental market in Italy is associated with “a clear decreasing trend in the length of the contracts”. As in the case of Italy, there appears to be a decline in contract duration in the Netherlands where short-term or liberal leases are increasingly adopted (Vranken et al 2021).

The increasing activity in land rental markets leads us to important questions about the duration of land rental agreements. In the USA, Bigelow et al (2016) illustrate the share of rented acres according to the duration and show that the majority of the rental area is associated with rental periods of seven years or greater duration. The research identifies notable differences in duration between non-farming landowners and farm-operator landowners with the former involved in contracts of longer average duration. Deaton et al (2018) find that long-term rental arrangements are associated with long-term conservation practices such as cover crops. Elsewhere, Hüttel et al (2016) used data on land lease duration to study the so-called term structure of land lease agreements in the German region of Saxony-Anhalt. However, few econometric studies have explored the determinants of contract duration in developed countries.

The type of farming system can influence the choice of land tenure and duration of possible rental agreements. In Ireland, tillage farming has historically occupied more rented land than dairy farming (Conway 1986; O’Neill and Hanrahan 2012). However, the production cycle is shorter in tillage farming and this may lead to rental agreements with a shorter duration. The profitability of dairying has surpassed tillage in the last decade (Loughrey et al 2022) and this shift in relative profitability also influences the relative demand from each activity for long-term leases. There appears to be an increase in the importance of contractualization for dairy farming in Ireland. This trend is further evident in the UK (Thorsøe et al 2020). In addition to the traditional agricultural activities of livestock and cereal production, there remains an interest among potential tenants for acquiring agricultural land to undertake other agri-environment related activities including forestry and wind farming (Van Rensburg et al 2015).

Much of the recent literature about the agricultural land rental market in Ireland is concerned with the decision of individual farm holders to participate in agricultural land rental markets or with the determination of land rental prices (O’Neill and Hanrahan 2012; O’Neill and Hanrahan 2016; Geoghegan et al 2021). Elsewhere, research emphasizes the potential importance of non-price conditions in the design of rental contracts. For instance, Adenuga et al (2021) emphasize the importance that tenants and landlords retain the ability to adapt to change, the ability to access new schemes, improve productivity and contribute to structural change. In particular, Adenuga et al. identify the possible role of a break clause in allowing the parties in the land contract “to review performance in relation to compliance with the terms of the lease”.

The family farm is the dominant type of farm holding in Irish agriculture. At the same time, there is evidence of family farms changing their legal status from sole trader to incorporated status and this development is documented in various reports (IFAC 2019), but is absent from the academic literature. Farm partnerships are also growing in popularity with approximately 3,000 registered farm partnerships in Ireland (Teagasc 2020). The legal status of potential tenants could play some role in influencing the demand for agricultural land leases, in terms of both price and duration. In addition, the expansion of tax incentives is motivating landowners into supplying more land to potential tenants (Geoghegan et al 2017). There is official evidence of an increase in uptake of tax incentives in relation to the leasing of agricultural land (Revenue 2021).

This paper is concerned with providing a deeper understanding about the determinants of contract duration during this current period of modest but important change for agricultural land tenure in Ireland. The research is based on the agricultural land lease market in two NUTS 3 regions in Ireland, i.e. the West region and the South-East region. These two regions are selected due to their different agricultural conditions. Agriculture in the West region is dominated by small-scale cattle and sheep livestock farming where economic viability is relatively low and where farm households rely significantly on off-farm sources of income. In the South-East region, the economic conditions are more favourable for dairy and tillage farming with higher levels of economic viability. The 2016 Farm Structures Survey provides the most recently available statistics about the area of agricultural land in each region and shows that the West region accounts for 17.4 per cent of agricultural land (excluding Commonage) while the South-East region accounts for 15.6 per cent in Ireland. These two regions together account for approximately one-third of the agricultural land area in Ireland. Research about the land rental market in these two regions can therefore provide a useful guide to the situation in the Republic of Ireland as a whole.

The research draws on the transaction cost economics approach and follows recent studies using transaction level data to analyse farmland markets including (Hüttel et al 2013; Seifert et al 2021) and a sparse literature exploring the influence of institutions on rural land markets (Needham et al 2011; Woestenburg et al 2014). Allen (1991) refers to Coase (1937) where transaction costs are defined simply as the “the cost of using the price mechanism”. In the context of the land rental markets in Ireland, the costs of using the price mechanism are arguably higher in the case of short-term informal agreements relative to long-term leasing. The paper performs an econometric analysis of the factors affecting the duration provision of (a sample of selected) agricultural land lease contracts in Ireland. The research represents an empirical test of the transaction costs economics (TCE) theory with analysis of the factors affecting the duration provision of (a large sample of selected) agricultural land lease contracts in Ireland. The research is undertaken to assess how (and how much) contract provisions affect the contract duration and whether or not the long-term contract is a transaction cost minimizing efficient structure. The authors attempt to interpret the results under a TCE framework and policy perspective.

## Policy framework

In Ireland, the policy framework in relation to agricultural land is evolving. However, this policy framework remains highly influenced by cultural and historical factors. These cultural and historical factors are reflected in the Irish constitution where "Policy framework" section of article 45 provides guidance to policymakers on the direction of social policy and stipulates “That there may be established on the land in economic security as many families as in the circumstances shall be practicable” (Constitution of Ireland 1937). For much of the twentieth century, the Irish economy remained highly dependent on agriculture (Honohan and Walsh 2002) and this goal heavily shaped agricultural land market policies (Sheehy 1982). In the twenty-first century, the policy framework has shifted to some extent to place more emphasis on land mobility and structural change in the farming economy. However, the policy concern for small farmers is still evident in the national strategy for the Common Agricultural Policy with references to farm viability and the survival of farms in areas of natural constraint (DAFM 2021). Farmers in Ireland maintain a strong cultural affinity to their land (Ryan and O’Donoghue 2016; Geoghegan et al 2021) and this undoubtedly continues to constrain the degree of land mobility.

Throughout most of the twentieth century, policy efforts favoured the ownership of farmland by owner occupiers as opposed to tenant farmers (Conway 1986). In the early twentieth century, the institutional framework was already in place to support such goals. In particular, the Irish Land Commission established under the Land Act in 1881 implemented nationwide land structural reforms, which continued after independence in 1922. Land rental market activity was restricted as the leasing of land (excluding 11-month conacre lettings) was subject to the express permission of the Land Commission (Conway 1986). The Commission was abolished in 1984 with modest increases in land rental activity occurring over subsequent decades (Swinnen et al 2016). Despite the abolition of the Land Commission, the dominance of short-term informal rental agreements remained for some time. Those landowners participating in land rental markets tended to continue operating under the “conacre” system by renting out land for one production cycle of 11 months with the possibility of yearly extensions.

The policy framework evolved to place more emphasis on land mobility and policymakers increasingly sought to better incentivise landowners into letting out land. In 1985, tax incentives were introduced to make rental income exempt from income tax up to approximately €4,100 per year (Euro purchasing power in 2021) with a condition that the land lease be of a minimum five years duration. These exemption limits increased substantially over time with variation according to the lease duration. For instance, the exemption limit for a five-year lease increased to €18,000 per annum in 2015 with higher limits for longer durations (Geoghegan et al 2021). The popularity of long-term leasing contracts only increased substantially from 2015 onwards (Revenue 2021). The policy framework shifted sufficiently to promote the emergence of long-term leasing on a significant scale and this is likely to manifest in a larger rental share at the national level.

Despite the apparent shift in the policy framework, the agricultural land market remains one of the least regulated in the European Union (Vranken et al 2021). However, some regulations have emerged in recent times. For instance, the 2011 Property Services Regulatory Act stipulates that rental agreements should be registered with the PSRA in circumstances where an auctioneer is engaged in the transaction.^1^ The PSRA is the statutory body in Ireland, which is responsible for licensing and regulating the property services sector. The PSRA maintains a Commercial Lease Register (including agricultural land leases) which details information about all commercial leases entered into since 1 January 2010.

More recently, landowners and farmer tenants in Ireland have faced increasing risk and uncertainty. Negotiations in relation to the Common Agricultural Policy have involved some degree of uncertainty and it is likely that this uncertainty increased in 2018 and 2019 as the 2014–2020 CAP programme reached the initially planned end date. Indeed, the link between the use of the land and the EU subsidy payments, introduced by the midterm review of CAP, and the re-negotiation of 2013, in which farmers who had leased out all of their land were not considered to be “active” farmers, may induce landowners and tenants to be cautious in making decisions that could put their future subsidies at risk (Geoghegan et al 2017). Therefore, the planned CAP reform and related information that landowners and tenants may retrieve from the regulatory proposals may affect decisions on contract duration. The definition of an active farmer is discussed in D’Andrea and Lironcurti (2017) while Guastella et al (2021) discuss the implications of farm subsidy reform on the value of land rental contracts.

In June 2020, the European Council reached an informal deal with the European Parliament on the extension of the CAP programme until the end of 2022 and a political agreement was reached in November 2020 on the transitional rules for 2021 and 2022 (European Commission 2020). The extension of the CAP programme until the end of 2022 has delayed the impact of any proposed reforms on agricultural land rental markets. Apart from the questions relating to the “active farmer”, the potential CAP reforms can influence land rental duration in a number of respects. For instance, the tendency for crop producing farmers to have a low participation rate in agri-environment schemes has been linked to a preference towards short-term rental agreements (DAFM 2021, p. 502).

### Theoretical and empirical background

**Fig. 1:**
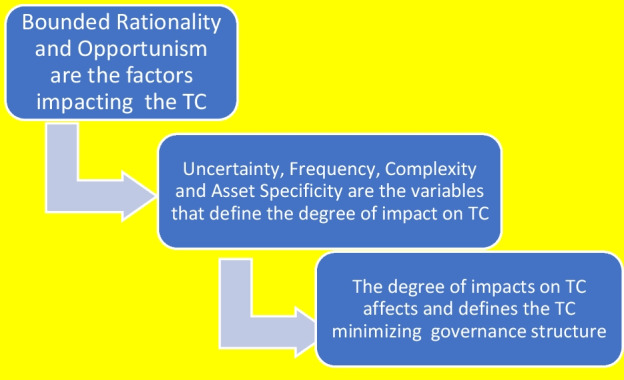
Transaction costs and governance structures. *Source* our elaboration

According transaction costs economics (TCE), the transaction is the basic unit of analysis where the transactions costs (TC) are the costs of negotiating, monitoring and governing exchanges.^2^ In Williamson’s model (1985), in fact, opportunism and bounded rationality are the two main drivers that impact and generate the transaction costs. The degree of intensity of the TC is affected by three main characteristics of the transaction: complexity (including frequency), asset specificity and uncertainty. TCE acknowledges different governance structures as a results of efficient TC minimization. This process is shown in Fig. 1.

The intensity of the TC (high, medium and low, expressed in qualitative terms) affects the governance structure, within which the transaction is organized. For instance, a high degree of complexity, asset specificity and uncertainty generate high TC. This, in turn, will drive the transaction organization towards vertically integrated governance structures to save TC. On the contrary, a low degree of complexity, asset specificity and uncertainty generate low TC. This will spur the transaction organization towards (spot) markets. In the middle of the two extremes exist a combination of the three key variables, with different degrees of impact on TC and, consequently, a plethora of governance structures that Williamson names “hybrid” and that span from cooperatives, to consortia, from long-term contracts to franchising contracts, just to mention a few.

Long-term contracts are hybrid structures, within which transactions are organized for efficient TC minimization. In the mainstream of transaction costs economics (TCE), it is a well-known theoretical result that the benefits of long-term contracting increase with the asset specificity required to undertake the transaction (need to secure the transaction) and decrease with the complexity and the uncertainty of the transaction (need for a flexible contract). TCE define asset specificity as “durable investments that are undertaken in support of particular transactions” (Williamson 1985, p.55). If these investments have lower value outside the transaction (exchange) undertaken, actors should preserve their relationship to make them profitable and save them to opportunistic behaviour of the exchange partner. Williamson (1985, 1996) show how the contract forms and the governance structure may be aligned with the degree of specificity. For TCE, a high asset specificity is one of the main reasons for actors to enter into a long-term contract.

From an empirical perspective, the study follows a well-recognized body of the literature, starting with the seminal papers by Joskow (1987) and Crocker and Masten (1988), and including the contributions of Saussier (2000), Masten and Saussier (2002), Onofri (2008). Masten and Saussier explain that the neoclassical and transaction cost approaches have dominated the economic analysis of contracting and that there is some overlap in the structure of the decision-making under these two approaches. In this research, we draw from the transaction cost approach to provide further understanding about the determinants of contract choice. While the standard neoclassical model emphasizes the roles of uncertainty and incentive alignment, the transaction cost approach views contracts as devices for reducing wasteful activities around the negotiation of surplus and the structuring of ex post adjustments (Masten and Saussier 2002). Transaction costs include those associated with information, the negotiation and writing of contracts and their supervision, enforcement and resolution in case of conflicts (Williamson 1985). Transaction cost economics is particularly concerned with the specificity of investments as the risk of opportunistic behaviour increases with the level of transaction-specific investments (Delmas and Marcus 2004). A formal contract specifying, in advance, the terms and conditions for future exchanges provides an appropriate mechanism to overcome the expropriation of specific investments (Hart and Holmstrom 1987; Joskow 1985, 1987) and the risks of incomplete contracts (Fares and Saussier 2002; Hart and Moore 2007).

### More specifically

#### H1

Transactions characterized by high asset specificity lead to long-term contractual relationship.

The bargaining of the long-term may be faced with the uncertainty of the transaction environment. High uncertainty makes impossible for actors (bounded rationality) to define in advance the events that may affect the profitability of their transactional relationship and consequently exposes them to more or less important transaction costs depending on the asset specificity. Crocker and Masten (1988) and Brickley et al. (2006) highlighted, in the context of inter-firm exchanges, that uncertainty raises the initial costs of writing contingent contracts, thus encouraging shorter lengths. Transactional partners who are interested in long-term contingent contracts incur not only significant “ink costs”, but also important search and negotiation costs to obtain informational and negotiating advantages over their partners (Klein 2002).

#### H2

A higher environmental uncertainty increases the probability of choosing short-term contractual relationship.

High environmental uncertainty requires regulating all possible circumstances that can impact the transaction at stake. However, writing complex contracts can be costly and these costs bring about the adoption of imperfect agreements that only offer limited protection against opportunism. As a result, an emphasis has been put on short-term relational contact rather than long-term contract (Mulherin 1986; Klein 2002). By the way, particularly excessively complex contracts may undermine the individual’s voluntary willingness to cooperate and induce opportunistic costly behaviour (Frey 1997). In addition, when asset specificity increases, contracts become complex because as the buyer enhances the features of his contract to (i) mitigate possible opportunistic behaviour by the seller (Klein 1996, 2000); (ii) avoid over-dependence on the seller (Anderson 1985); and (iii) be flexible to respond to changes in the environment of the transaction (MacLeod 2000). The search by contractual partners for savings on transaction costs linked to the complexity of their contract leads them to short-term contracts.

#### H3

A higher transactions complexity and transaction frequency decrease the probability of choosing long-term contractual relationship.

The choice of the duration of the contract also depends on the trade-off that the parties can find on the attributes of their transaction to minimize its transaction costs and optimize its profiles. This might occur because a higher frequency of payment or exchanges, for instance, can decrease transaction uncertainty and allow for a quick adaptation to changing circumstances with respect to the initial date of the contract.

On the one hand, long-term contracting provides benefits, like the reduction in the cost of repeated bargaining and bigger willingness of transactors to take actions, whose value depends on the other party’s performance, thus better adapting to complexity. On the other hand, there are drawbacks on long-term contracting, stemming from the costs of anticipating, devising optimal responses to and specifying future contingencies (formation costs); and from the losses associated with efforts to enforce, evade or force a renegotiation of the contract’s terms and the “maladaptation” costs of failing to adjust to changing circumstances (execution costs).

In this perspective, contract terms align ex ante marginal incentives and prevent wasteful efforts towards ex post redistribution of existing surplus. For instance, long-term contracts that specify the terms and conditions for future transactions ex ante represent a remedy for ex post performance problems. In this perspective, contract duration is a key synthesis indicator to understand the mechanisms that drive the parties’ reciprocal incentives and surplus redistribution of value, in the context of Irish agricultural contracts.

Although a complete literature review goes beyond the scope of this paper, many recent studies in agricultural economics have explicitly followed the transaction cost approach. For instance, Bakucs et al (2013) use this approach to study contract choice among Hungarian farmers in the supply of milk to processors. Valentinov (2007) use the TCE approach to study the role of cooperatives in agriculture. Traversac et al (2011) use this approach to study the decision of wine producers in France to enter into direct selling. Wen and Chatalova (2021) adopt the TCE approach to study the impact of transaction costs on farm size in Germany.

Specifically, this research will further our understanding regarding the determinants of contract duration in agricultural land rental markets. Few econometric studies have addressed the determinants of contract duration in agricultural land markets in developed countries. The exceptions include Bandiera (2007) and Ackerburg and Botticini (2002) both of which were concerned with the determinants of contract form in Italian agriculture during the distant past. Marks-Bielska (2013) outlined some of the relevant theory, but there is a void in terms of the economic literature dealing with the determinants of contract duration for land markets in today’s agriculture.

Elsewhere, Ilbery et al (2010) use qualitative methods to explore the changing landlord–tenant relationship in England and consider the role of legislation including the Agricultural Tenancies Act of 1995 involving the introduction of the Farm Business Tenancy (FBT) and a move towards more formal contractualization. The authors question the success of the FBT concluding that most FBT agreements have covered relatively small amounts of land under relatively short duration. The research identifies spatial patterns in the adoption of the FBT with greater adoption in the south relative to the north of England where traditional forms of leasing remain prevalent.

## Research methods

The section presents the data, the selected testable implications and empirical strategy.

### Data

The dataset for this research is based on a large sample of land leasing transactions from the Property Services Regulatory Authority (PSRA) in Ireland. The pooled cross-sectional dataset contains information about transactions in the West and South-East NUTS 3 regions from 2013 to the onset of the COVID-19 pandemic in March 2020. The original data contained 3,644 transactions for agricultural land lease contracts in Ireland. However, 105 of these observations were deemed unusable mainly due to the absence of information about the parcel size or in a small number of instances where the declared parcel size is less than one acre, i.e. 0.405 hectares. This leaves a final dataset of 3,539 transactions.

Each contract contains various types of information and provisions, spanning, among the others, on the agricultural activities performed by the tenant, rental value; dimension of the parcel; contract duration; payment frequency; type of tenant (sole trader or institution), geographical location of the parcels (county location), date of negotiations and entry into force of the contracts, a set of provisions that affect performance (e.g. legal notice, breaking clauses, insurance and so on).

The amount of agricultural land in medium- or long-term leases increased strongly from 2014 onwards and from quite a low base. This could be attributed to the abolition of milk quota and the expansion of tax incentives in 2015. Ireland is identified as the EU member state with the largest percentage increase in milk production since the abolition of the milk quota system (Lapple and Sirr 2019) with an approximately 25 per cent increase in raw milk production from 2012–2014 to 2015–2017 (Cele et al. 2021). Due to the land-based nature of dairy farming in Ireland, this large increase in production required a significant increase in the land available for dairy farming via land purchase or land rental.

In particular, some caveats need to be highlighted. We do not specify whether or not the land lease contract is completely new. However, we expect that a large majority of these contracts are new contracts, i.e. not previously agreed under a formal written contract. Some of these parcels may previously have been rented on an informal basis with just one year duration i.e. under the conacre system (Patton and McErlean 2003; Geoghegan et al 2017). The amount of agricultural land in medium- or long-term leases increased strongly from 2014 onwards and from quite a low base. This could be attributed to the abolition of milk quota and the expansion of tax incentive in 2015. The transaction data largely contains information about land lease contracts of at least five years duration.

Table 1 reports descriptive statistics for selected variables. The average annual rental value is €7,538 with a standard deviation of €9,690 and a maximum rental value of €100,000. Interestingly, a small proportion of transactions have a zero value for the rent. This is not an unusual occurrence given that transactions may take place between relatives where a high degree of trust exists. Zero rent contracts are observed in all counties, but appear relatively more prevalent in counties Galway and Mayo where some land can be quite marginal.

**Table 1:** Descriptive statistics (*N* = 3,539)

Variable	Mean	Std. Dev	Min	Max
Annual rent	7,538.05	9,690.02	0	100,000
Duration (Months)	90.26	83.84	5	1,450
Leased land (Per Parcel)	21.83	32.50	0.2	1,195
Type of agricultural activity	Pasture (65.44%); tillage (33.40%); forestry (1.16%)			
County	Carlow (6.21%); Galway (19.56%); Kilkenny (19.73%); Mayo (13.20%); Roscommon (7.50%); Waterford (17.22%); Wexford (16.59%)			
Break clause	No = 95.41%; Yes = 4.59%			
Payment frequency	Annual (41.76%); biannual (37.74%); one instalment (0.55%); monthly (5.85%); other (14.36%)			
Tenant type	Individual tenant (75.46%); organization (24.54%)			
Rent review	No = 83.7%; Yes = 16.3%			
Notice period	No = 97.09%; Yes = 2.91%			

The average duration is 90.26 months with a standard deviation of 83.84 months. The minimum duration is five months and the maximum is 1,450 months. The average parcel size is 21.83 hectares with a standard deviation of 32.5 hectares, thus pointing to high variability between transactions. The minimum parcel size in the dataset is 0.2 hectares and the maximum is 1,195 hectares. There is some variability in the type of land use with tillage land accounting for 33.4 per cent of transactions, pasture accounting for 65.4 per cent and forestry with 1.16 per cent of transactions. Break clauses apply to just 4.59 per cent of transactions with most transactions having no evidence of this provision. Notice periods apply to just 2.91 per cent of transactions. Individual tenants have traditionally been the main source of demand for long-term land leases and account for 75.46 per cent of transactions. At the same time, organization tenants account for 24.54 per cent of transactions. Table 1 reports descriptive statistics for selected variables. Appendix 1 contains a description of the variables.

### Testable implications and empirical strategy

In our setting, the contract duration is a proxy that indicates a type of governance structure, as in Joskow (1985). Contract duration, measured in years, is a variable that proxies long-term contracts. The main hypotheses to be tested are that long-term contracts are a TC-governance minimizing structure for the land lease in Ireland. Following up the theoretical framework, illustrated in the previous section, our expectations are synthesized in Eq. (1)

1 $$\begin{gathered} ~{\text{Long term contracts }}\left( {{\text{proxied by Duration}}} \right){\text{ }} = {\text{ f}}\left( {{\text{Asset Specificity}};{\text{ Uncertainty}};{\text{ Complexity}};{\text{ Frequency}}} \right). \hfill \\ ~\quad \quad \quad \quad \quad \quad \quad \quad \quad \quad \quad \quad \quad \quad \quad \quad \quad \quad \quad \quad \quad \quad \quad ~\left( + \right)~\quad \quad \quad \quad ~\left( - \right)~\quad \quad \quad \quad \left( - \right)\quad \quad \quad \left( - \right) \hfill \\ \end{gathered}$$

A high level of asset specificity increases TC and, therefore, the contract duration since the asset needed for the transaction is not easily redeployable elsewhere. In our dataset, the asset specificity is represented by the “no concession^3^” variable that represent a physical and financial asset specific investment that the tenant should do (for repair, improvement and equipment purchasing and building) for the sake of contract implementation. Those investments would not be easily redeployable (at least not all) outside the transaction, therefore increasing TC and the contract duration.

High frequency of transactions decreases TC and contract duration. In our dataset, the frequency of transaction is represented by all variables describing the type of payment (monthly, annual, biannual and so on).

Complexity and uncertainty of the transaction increase TC. For this reason, the contract duration tends to be shorter in order to let the parties to adapt less costly to changing uncertain and complex circumstances. In our framework, “rent review”, “break” and “notice period” clauses represent the parties’ way to deal with uncertainty and complexity. The inclusion of these provisions in the contracts is expected to increase contracts duration.

Following up the testable implications, our modelling reasoning, and related choice of the empirical estimation method, has also to include the assumption that a set of provisions is predetermined and exists before contract, while another group of provisions are determined within the contract, together with the duration. For this reason, we operationalize the relationship between contract duration as follows:

2 $$\left( {{\text{Log}}\_} \right){\text{Duration}}_{i} = \, \alpha i + \, \beta_{1} Y_{i} + \gamma_{2} Z_{\rm i} + \, \varepsilon_{i}$$

where the dependent variable is contractual duration and is estimated in the logarithms. It is important to highlight that the duration of the contract is not expressed in discrete value. In particular, the empirical model, with the dependent variable in the logs, can be interpreted as a duration model, featuring log-normal hazards (see Onofri, 2008).

In (1), Yi indicates the endogenous variables, including terms of contract, and *Z*_i_ indicates instruments. In particular, we estimate a simultaneous equation model where the selected instruments (i.e. the geographical location of the parcel) represent variables determined before contracting. Instrumental variable approach has been used elsewhere in the field of agricultural economics under the TCE approach (Lu et al 2008; Daum et al 2021). The selected endogenous variables (i.e. inclusion of a breaking clause) represent provisions that are jointly determined within the contract and that are jointly determined with the contract duration and are proxies of asset specificity, uncertainty, complexity and frequency. The model includes a constant and the error term.

The estimated relationship is simply exploratory in nature, with the objective to assess how (and how much) contract provisions affect the contract duration, attempting to interpret the results in a TCE framework. We adopt the two-stage least squares (2SLS) estimation techniques for estimating with STATA 16 the relationship between contract duration and contractual provisions.^4^

## Results

Table 2 presents the econometric results that we have selected after many checks. Model 1 estimates contract duration as depending on rent; monthly payment; notice period provision; and no concession provision, individual tenant and a constant. Instruments for those variables are listed in Table 2 and represent a set of variables related to the land area, the land location and the year when the contract has been signed, are predetermined and existing before the contract and, in this way, affect the choice of the contract provisions and the contract duration. Selected instruments are the parcels area, the type of agricultural activity performed in the contract (tillage, forestry and so on); the geographical location of the parcel (being the county where the parcel is located, also a proxy indicator for the economic milieu, within which the contract is negotiated, signed and enforced); the year when the contract has entered into force.

**Table 2:** 2SLS estimation

Instrumental variables	Model 1	Model 2
(Log)Rent	0.07 (0.46)	–
No rent	–	− 0.52 (0.56)
Break clause	–	1.03*** (0.02)
Monthly payment	− 0.81* (0.30)	–
Individual tenant	− 0.48*** (0.03)	− 0.72*** (0.07)
Notice period	0.83* (0.21)	–
No concession	3.98*** (0.6)	–
Constant	0.08 (0.54)	4.82*** (0.08)
Diagnostics	Wald chi2 (5) = 26.09 Prob > chi2 = 0.001 Durbin–Wu–Hausman test of endogeneity = 17.94 (*p* = 0.0030) 3.59 (*p* = 0.0030)	Wald chi2 (3) = 32.21 Prob > chi2 = 0.0000 Durbin–Wu–Hausman test of endogeneity = 24.87 (*p* = 0.0000) 2.78 (*p* = 0.0000)

Dependent Variable: (Log)Contract Duration

Instruments: logarea tillage forestry Galway*size Kilkenny*size Mayo*size Roscommon*size Waterford*size Wexford*size year_2013 year_2015 year_2016 year_2017 year_2018 year_2019

^***^0.1% statistical significance; *0.05% statistical significance. Standard errors in parentheses

Model 2 estimates contract duration as depending on a set of variables, in the case that the contract does not involve a monetary payment for the land lease. Selected Instruments are the same as in Model 2 for the sake of comparability.

Results for model 1 show that if the contract includes a notice period clause and a “no concession” provision,^5^ then the contract duration extends to longer periods. The higher the rent the tenant has to pay, the longer the contract (a 1% increase in the contract rent generates a 0.07% in the contract duration). If the tenant is an individual (and not an organization, for instance an incorporated farm or partnership), the contract duration is shorter. Monthly payments negatively impact the contract duration.

When the land lease contract does not include a rent payment, as in Model 2, the contract duration is shorter. The inclusion of a break clause extends the contract duration. Also in this case, if the tenant is an individual (and not an organization, for instance an incorporated farm or partnership), the contract duration is shorter.

The overall diagnostic of the model indicates that estimates are correct; therefore, the explanatory power of the selected empirical specification is robust. In particular, the Durbin–Wu–Hausman test of endogeneity rejects the null hypothesis that variables are exogenous since p values are less than 0.05.

## Discussion

The results are consistent with transaction costs economics theory. The parties have the incentive to mitigate long-term contract inflexibility through the negotiation of ex ante bargained terms and conditions, with provisions that allow for contingent adaptation. For instance, a zero rent may be balanced by the possibility to exit the contract, through the definition of a shorter time horizon. At the same time, long-term contracts can increase the risk of ex post maladaptation, thus creating demand for processes that enable adaptation over the course of long-term exchange. This motivates the inclusion of provisions such as break clauses and notice periods. However, long-term contracts can increase the risk of ex post maladaptation, thus creating demand for processes that enable adaptation over the course of long-term exchange.

The provisions that regulate the frequency of the transaction (monthly payment) negatively affects the contract duration. This might occur because a higher frequency of payment (monthly) can decrease uncertainty and allow for a quick adaptation to changing circumstances with respect to the initial date of the contract. Under transaction cost economic theory, lower payment frequency increases transaction costs, and as predicted by this theory, this increases the contract duration/favours the adoption of longer terms contracts. In this perspective, the contracts that include provisions for a higher frequency of payment tend to be shorter.

The contracts with break and notice period provisions are longer. The notice period is an adaptation (to uncertainty and changed contingencies) are therefore more procedural than a break clause. The notice period, for instance, can vary from one month to six months. On the contrary, the requirement to "re-appropriate the resource" is more immediate and quicker with a break clause. Those results show that the provisions are used as ex ante contractual remedies to possible ex post maladaptation, due to unexpected contingencies or even opportunistic behaviour in two different situations (rent/no rent). This suggests a relationship between the drivers that generate TC (uncertainty, complexity, asset specificity, etc.) and the selected (possibly) transaction cost minimizing governance structure, even if expected signs differ from standard theory. Such results differ from theory-based expectations and testable hypotheses, but are validated by the literature. The seminal paper by Cheung (1969), for instance, mentions a special contractual arrangement, the “escape clause” in Chinese agricultural contracts with fixed rents. Those contracts explicitly clarify that, “in a famine year, rental payment shall be adjusted (downward) according to local custom” and “the (aforementioned) rental rate is subject to adjustment according to local”. Cheung then argues that with multiple “escape clauses” for tenants, fixed rent contract is no longer “fixed”; therefore, the contract provisions, including duration, can be renegotiated. Allen and Lueck (1992) find similar clauses in agriculture in the USA. Elsewhere, Maher (1997) for other industries observes that longer-term contracts are more likely to contain break clauses.^6^

In all estimated specifications, the results indicate that if the tenant is an individual (and not an organization, for instance an incorporated farm), the contract duration is shorter. A shorter contract implies a governance that is closer to “market” transactions and therefore lowering TC. This may occur because contracts with individuals are less complex than those with incorporated farms. TC are lower and contracts are shorter. In addition, incorporated farms account for less than one per cent of all farm holdings in Ireland (CSO 2018). Another interpretation is that the tendency for organization tenants to partake in contracts of longer duration could be due to the ability of large farm companies to undertake the transaction costs (Buduru and Brem 2007). The result appears very coherent with the uncertainty generated by the CAP payments. Individual tenants are more likely to be the recipients of CAP payments, which are decided on triennial basis. This increases uncertainty (and TC) that parties may be willing to overcome/minimize by signing contracts with a shorter duration. In addition, there could be less uncertainty around farm succession issues on incorporated farms where viability is probably higher. An increasing number of farm families engaged in dairy farming are switching to incorporated status to reduce tax liabilities (IFAC 2019). Farm partnerships are popular among dairy farmers. Both farm partnerships and incorporated farms are much more prevalent in the South-East region relative to the West region where dairy farming is much less prominent. Apart from dairy farming, organization tenants are involved in other farming enterprises including horticulture, forestry, pig farming, poultry and wind farming.

Finally, the no concession clause provision, as a proxy of financial and or physical assets specificity increases the contract duration, as predicted by the theory. If the tenant must incur in specific investments for improvements and cannot obtain a rent reduction in exchange, then higher TC, generated by asset specificity is minimized within a longer contract governance structure. In addition, the motivation for the inclusion of non-price provisions appears rational when CAP reform policies can potentially influence the land rental values and eligibility for subsidies. In this context, the inclusion of non-price provisions enables both the lessor and lessee to adapt to potentially changing policy circumstances.

Overall, the results indicate that the long-term contracts are not TC minimizing governance structures, but are highly influenced by a set of regulatory incentives (tax exemptions and the CAP subsidies) that may overwhelm the boundaries of private bargaining. This may explain why the estimated coefficients for the contractual variables (selected and bargained upon by the parties) are not statistically significant and why the most "incisive" clauses are those that represent the "way out" (notice and break).

## Conclusions

The study has performed econometric analysis of (selected) factors that affect contract duration, with an application to agricultural land lease contracts in two regions in Ireland. Results corroborate TCE theory. Contracting parties, while negotiating upon duration, need to balance the need of securing a long-term horizon for the generation of mutual surplus from contracts execution and the need to guarantee flexibility to adapt to changing circumstances. Empirical findings support, in the case at issue, such a theoretical approach. The econometric results indicate that the presence of non-price provisions such as break clauses and notice periods increase the contract duration and provide the necessary flexibility for long-term contracts to be agreed and formally written.

Concurrently, the low adoption rates for these provisions indicates that insufficient attention may be allocated to their inclusion in written contracts. This conclusion is supported by the fact that the practice of long-term farmland leasing is a new activity for many landowners and tenant farmers in Ireland. There is likely to be a high degree of inexperience in the setting of these formal written contracts. In theory, such inexperience could play a number of roles. It could lead to more conditions such as break clauses and frequency of payment as both parties exhibit additional caution. However, inexperience could also manifest itself in fewer conditions with implications for transaction costs at a later stage and this appears to be the more likely representation of the Irish agricultural land market. There appears to be an important role for policymakers and relevant stakeholders in improving education for farmers and landowners in relation to the inclusion of non-price provisions within land rental contracts and therefore optimize the duration of contracts.

Furthermore, the statistically significant coefficients associated with variables that improve the flexibility of the contract allows us to argue that other factors (important for TC generation, but not included in the parties’ bargaining possibility set), such as the policy context and incentives (tax incentives and subsidies), may drive the decision-making of both tenants and landowners. Indeed, when the tenant is an individual farmer (more likely to be a recipient of agricultural policy support), we observe the tendency to reduce the length of the contract. This behaviour can be justified by the need to manage the uncertainty coming from the recursive reform of the CAP. However, further research is required to uncover the contribution of policy uncertainty towards contract duration.

This might imply that even if long-term contracts contain important TC minimizing provisions (break clause, notice clause, individual tenant), they do not necessarily represent the most TC minimizing governance structure. Policy instruments "imposed" by economic or regulatory reform can override the concern for TC minimizing provisions that may be expected to emerge from the parties acting rationally and independently. The abolition of milk quota and the expansion of tax incentives have undoubtedly influenced the decision of many landowners and tenants to enter into the land leasing market, but this may be occurring in the absence of sufficient consideration for the importance of TC minimizing provisions and the adoption of TC minimizing governance structures that are different from long-term contracts (TC are not administrative costs).

Until recently, most attention from researchers and policymakers with respect to the land rental market in Ireland has focused on the marginal economic gains from land market participation under the favourable conditions of rising land rental prices and expanding tax incentives. This reading of the land market is consistent with neoclassical economics where market equilibrium and the alignment of financial incentives are the main focus of the argument. This research shows the potential contribution of institutional economics under the TC approach where theory can be tested using administrative level data on complex aspects of land rental transactions, which are often ignored under standard economic theory. Obviously, the results (and suggested implications) are derived from an analysis that is based on data about transactions registered with the relevant regulatory authority. Some transactions may not be registered with the regulatory authority. Informal one year renting agreements are mainly excluded from the official register and these transactions lie outside the scope of the research.

In this perspective, further research might focus both on (1) analysing the intended use on the duration of the rental contract (agisment, conacre and so on) and (2) thinking, designing and comparing alternative governance structures (short-term contracts or other hybrid structures, like contracts that transfer the performance risks or coordinated agreements between the parties) that might better implement the Irish reform of the land lease. The duration of rental contracts, in fact, is regulated in some of the EUSCs, which influences the responsiveness of the rental market to agricultural policy changes. The length of rental contracts is regulated by the government in Belgium and France (with a contract duration of nine years minimum), the Netherlands (six years minimum) and Spain (five years minimum). In several EUSCs (e.g. France), the renewal/ inheritance of rental contracts is also regulated. The prevalence of land renting is typically higher in countries with strict rental market regulations, such as Belgium and France. In these countries, formal rental markets are stickier and the time lag is longer in adjusting to policy changes.

## Data Availability

The datasets used and/or analysed during the current study are available from the corresponding author on request.

## Appendix Table

**Table:**

Variable name	Variable name(s) in PSRA dataset	PSRA description	Further notes
Annual rent	am_average_rent	This section contains details of the average annual rent for the lease up to the first rent review period. If there is no rent review contained in the lease, this section contains the average annual rent for the entire period of the lease	
Duration (Months)	qt_lease_year qt_lease_months	This section of the form refers to the length of the lease in years. This section of the form refers to the length of the lease in months	The duration is calculated based on the two variables representing the number of years and number of months of the contract
Leased land (Per parcel)	qt_nonres_land	This section of the form refers to the amount of land (in hectares) that is the subject of the Agricultural Lease	In cases where there is no information on this variable, we use data on the land area of three land types, i.e. agisment, conacre and forestry (see next description below)
Type of agricultural activity	agAgistment agConacre agForestry	This section shows the area of the land of an agricultural lease that is used for Agistment (Pasture). This section shows the area of the land of an agricultural lease that is used for Conacre (Tillage). This section shows the area of the land of an agricultural lease that is used for Forestry	This variable is based on information from the three variables listed in column 2. There are three categories. Agisment, i.e. pasture Conacre, i.e. tillage Forestry
County	dc_county	This gives the name of the county in which the property is situated	There are 26 counties in the Republic of Ireland. This dataset includes information about land parcels in seven of these counties, i.e. Mayo, Galway, Roscommon, Waterford, Wexford, Carlow and Kilkenny
Break clause	BreakClauseParticulars	This section contains the details of whether or not there is a Break Clause in the lease, this is a yes or no question	
Payment frequency	Frequency	This section contains information on how often the rent is paid, i.e. weekly, monthly, quarterly, etc.	
Tenant type			
Rent review	rentReview	This section deals with a Yes or No question as to whether or not there was a rent review for the property	
Notice period	NoticePeriod	This section contains the details of whether or not there is a Notice Period required in relation to the Break Clause. This is a Yes or No question	

## Abbreviations

TC: Transaction costs
TCE: Transaction costs economics
2SLS/3SLS: Two-/three-stage least squares
CAP: Common Agricultural Policy
PSRA: Property Services Regulatory Authority
FBT: Farm business tenancy

## Footnotes

1 Property Services (Regulation) Act 2011 http://www.irishstatutebook.ie/eli/2011/act/40/enacted/en/html

2 The section draws from the work of Oliver Williamson (1967, 1979, 1985).

3 The variable “concession” refers to concessions given by the landowner to the tenant. It appears that some tenants are given the benefit of reduced rent if they undertake fencing or other improvements around the farm.

4 Some technical clarifications are required. With respect to potential self-selection problems, it is the case that the dataset only contains information about observed land rental transactions and mainly transactions of at least five years duration. However, the dataset has the advantage in that it is regulatory data. Under the 2011 Property Services Regulatory Act, all rental agreements must be registered with the Property Services Regulatory Authority (PSRA) in circumstances where an auctioneer is engaged in the transaction. Agreements must also be registered with the PSRA to permit landowners to avail of certain tax incentives. The dataset therefore contains a large share of the actual transactions taking place. Informal rental agreements (~ one year) are likely to have a lower value per hectare and are mainly excluded from the data. The addition of informal land rental agreements could lead to a different result for the relationship between rental price and duration. However, we are focusing on the duration of land rental agreements for formal contracts, which tend to be five years or more. On the supply side, there is likely to be some self–selection as older farmers and less viable farms are more likely to lease out their land. In some cases, the land may have been inherited and there is limited interest in farming. On the demand side, there is likely to be self-selection for younger and more profitable farms. Recent reports indicate that this is one of the main reasons for the letting out of agricultural land in Ireland (SCSI/Teagasc 2021). Some farmers have decided that there is sufficient owned land and these farms therefore do not appear on either side of the transactions. Regarding the treatment of endogeneity problems, we are mainly relying on transaction level data with limited information about the farmers’ characteristics. The variables, though, are selected following rigorous economic thinking and checked statistically. For instance, we expect that the location variables are good instruments because they do not have a direct influence on the duration. Due to differences in land quality, the county location can influence the land rental price and this is one of the main endogenous variables.

5 The variable “concession” refers to concessions given by the landowner to the tenant. It appears that some tenants are given the benefit of reduced rent if they undertake fencing or other improvements around the farm.

6 The low proportion of contracts involving a break clause may indicate a level of inexperience in contract formation. In Ireland, tax incentives have promoted the decision of many landowners and tenants to enter into a contract with a minimum of five years duration. However, this does not necessarily lead to the best contract design given that inexperience and a lack of consultation with expert advice could lead to the absence of potentially important provisions such as the break clause or notice period.
